# Living on the edge: a longitudinal study of *Anopheles funestus* in an isolated area of Mozambique

**DOI:** 10.1186/1475-2875-12-208

**Published:** 2013-06-17

**Authors:** J Derek Charlwood, Nelson Cuamba, Elsa VE Tomás, Olivier JT Briët

**Affiliations:** 1 DBL Centre for Health, Research and Development, University of Copenhagen, Fredriksberg, Denmark; 2 Laboratory of Entomology, National Institute of Health, PO Box 264, Maputo, Mozambique; 3 MOZDAN (Mozambican-Danish Rural Malaria Project), PO Box 8, Morrumbene, Inhambane Province, Mozambique; 4 Swiss Tropical and Public Health Institute, Socinstrasse 57, PO Box, CH-4002, Basel, Switzerland; 5 University of Basel, PO Box, CH-4003, Basel, Switzerland

**Keywords:** *Anopheles funestus*, Aestivation, Survival, Island

## Abstract

**Background:**

Understanding the survival strategies of malaria vectors at the edges of their distribution, where they are under stress from environmental conditions, may lead to the development of novel control techniques and may help predict the effects of climate change on these mosquitoes.

**Methods:**

The population dynamics of an isolated population of *Anopheles funestus* from the peninsula of Linga Linga in southern Mozambique was studied over a period of 104 weeks from March 2009 to May 2011 by 917 light-trap and 390 exit collections, mostly in an area close to a seasonal pond.

**Results:**

Over the sampling period, 3,684 *An. funestus* females were caught. Densities decreased with increasing distance from the pond. In 2009 and 2010, a single annual peak in *An. funestus* density coincident with the single annual peak in rainfall was observed, but a clear population peak was absent during the first 21 weeks of 2011. In between population peaks, *An. funestus* remained present at low densities. In light trap collections, the proportion of gravid mosquitoes was significantly higher during the ‘low season’ (the period between peaks) than during the peak season (RR = 4.3, p<0.001). In contrast, in exit collections, the proportion of gravid mosquitoes was significantly lower during low season than during the peak season (RR = 0.64, p<0.01). Also, in light traps, the proportion of part-fed females was higher during the low season than during the peak season (RR = 4.5, p<0.001), whereas this was inversed for engorged females (RR = 0.46, p<0.05).

Thirteen out of 289 (4.5%) *An. funestus* tested positive in the sporozoite ELISA. The proportion of sporozoite positive females was higher during the low season (6.25%, six out of 96) than during the peaks (3.63%, seven out of 193), but this difference was not significant.

**Conclusions:**

It is suggested that a proportion of the mosquito population may become gonotrophically discordant during the long dry season resulting in enhanced mosquito survival and sustained malaria transmission.

## Background

The dynamics of malaria vectors are particularly interesting at the edges of their distribution since it is there that they are furthest from the centre of their niche and it is there that they are likely to be under stress from environmental conditions. At the edges of their distribution, species are likely to form meta populations, that is, small isolated populations each of which may temporarily become extinct, the habitat remaining empty until recolonized. By focusing on the ecology of mosquitoes in extreme conditions, the reasons for extinction might be determined which could inform strategies to eliminate the mosquito over wider areas. Understanding the survival strategies of mosquitoes in these situations may also help predict the effects of climate change on these vectors and may also lead to the development of novel control techniques. Rather than using a blanket approach, targeted approaches that enhance the effects of natural stresses may be useful.

One way for mosquitoes to cope with stresses of temporarily unsuitable conditions is to aestivate (during hot and dry periods) or hibernate (during cold periods). In doing so, they become gonotrophically discordant and take multiple blood meals per egg batch. To paraphrase Tolstoy [1], all gonotrophically concordant populations of anophelines are the same, whereas all gonotrophically discordant populations are discordant in their own way. The term ‘gonotrophically discordant’ was first introduced by Roubaud [2] to describe multiple blood-feeding in hibernating *Anopheles atroparvus* from northern Europe. Hibernation occurs in temperate zones, induced by short day lengths. In hibernating populations, all mosquitoes are affected, and gonotrophic discordance is complete. Aestivation, which has been discussed in detail by Washino [3], occurs in torrid zones (tropics). Not all mosquitoes in such populations aestivate, and gonotrophic discordance is not complete. Gonotrophic discordance of this sort has been described among *Anopheles maculatus*, *Anopheles culicifacies*, *Anopheles annularis* and *Anopheles aconitus* from India and Southeast Asia [4-6].

In Africa, studies on gonotrophic discordance have largely been limited to members of the *Anopheles gambiae* complex. At the extreme north of their distribution in the Sudan, *Anopheles arabiensis* can aestivate: during the long dry season, adult females rest inside houses or *turkels* and are prone to take small blood meals for survival purposes without developing more than a single egg batch [7]. Thus, they enter the dry season as young unfed females and end it as gravid ones. Because they take multiple blood meals per egg batch, even low numbers of such mosquitoes pose a threat since they survive for much longer than the extrinsic cycle of the parasite, hence infection rates may be high. However, in the presence of adequate breeding sites, such as the spill from water tanks in refugee camps, the mosquitoes continue to breed normally [8,9]. Consequently, for normal breeding, the availability of water is a critical component of their dynamics.

Holstein [10] considered that the *An. gambiae* s.l. found resting in the dry season in Upper Volta existed in a state midway between gonotrophic discordance (or disassociation) and gonotrophic concordance, developing eggs but blood-feeding whenever the opportunity arose. In the Sudan, three-quarters of the gonotrophically discordant *An. arabiensis* sampled by Omer and Cloudsley-Thompson [7], all of which were nulliparous, contained blood. More recently, Lehmann and colleagues have described a similar aestivation phenomenon occurring in *Anopheles coluzzi* (previously known as the M form of *An. gambiae *[11]), with one marked specimen being collected seven months after initial release [12,13]. They considered that the rise in density five days after the first rain was due to aestivating mosquitoes emerging from their resting sites. Neither the sympatric *An. gambiae* (previously known as the S form of *An. gambiae*), nor *Anopheles funestus*, nor, surprisingly, *An. arabiensis*, aestivated in this way. Such an explanation for the rapid re-appearance of mosquitoes is also mentioned by Muir [14]. Gillies [15,16] observed a similar phenomenon in *An. gambiae* in Muheza, Tanzania, but did not ascribe it to any cause. Should it have been due to aestivating insects, then this would imply that the behaviour is possible in all freshwater members of the *An. gambiae* complex, but that each species does or does not aestivate according to local conditions. Thus, aestivation was not observed in vectors in the semi-arid Kilombero Valley in Tanzania. Here, during the rainy season, rain-fed rice fields provide suitable breeding sites, and at the start of the long dry season, the vectors appeared to retreat into separate refugia where they were locally common: *An. gambiae* occurred close to the forested escarpment of the Uduzungwa Mountains where rainfall was over 200 mm/annum and where artisanal irrigation was practiced, *An. arabiensis* occurred at the margins of the Kilombero River (where it bred in animal footprints), and *An. funestus* was found in places where seasonal rivers had remained as substantial shaded ponds, without evidence of any form of gonotrophic disassociation [17] This makes it difficult to generalize the life-history strategies adopted by different species in different areas. In each of the situations where aestivation has been reported, however, one of the factors that characterized the mosquitoes was an exceptionally long survival time.

The one instance of gonotrophic discordance recorded in *An. funestus* was in hibernating rather than in aestivating mosquitoes: Leeson in 1931 ([18] quoted in [19]) found gravid females overwintering in cracks in rocks and riverbanks in Zimbabwe. Once temperatures rose, they left these sites, oviposited and died. Little is known about the biology of *An. funestus* in hot and dry environments and the threat that it poses there in terms of malaria transmission. Such environments may increase as a result of global warming with the associated reduction in rainfall in southern Africa [20]. One such hot and dry environment where continuous breeding is not possible, but where malaria is endemic and perennial, is the Linga Linga peninsula in southern Mozambique.

Here, two years of sampling are described and it is suggested that, in the absence of rain, *An. funestus* in Linga Linga is present for long periods, largely as low-density, gonotrophically discordant, females, which are effective vectors of malaria.

## Methods

### Description of the study site

**Figure 1:**
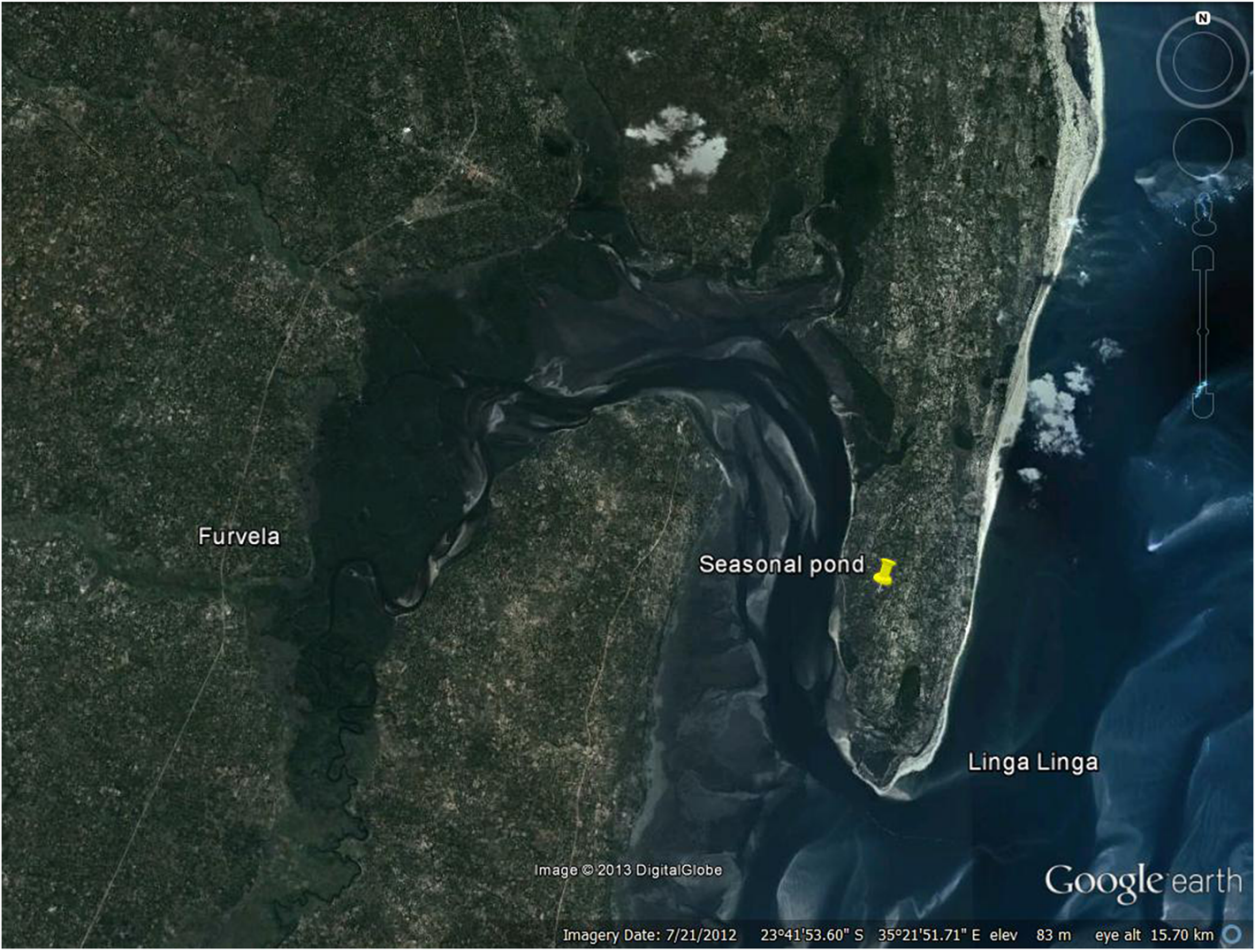
Location of Linga Linga relative to Furvela.

Bordered on one side by the Indian Ocean and on the other by Morrumbene Bay, the approximately 2 by 7 km peninsula of Linga Linga (23.7°S, 35.4°E) (Figure 1) is a sandy finger of land in southern Mozambique, opposite the capital of Inhambane Province and 8 km by boat from the district capital, the town of Morrumbene, which can also be reached by a 23-km sand road. There is an area of uninhabited bush, circa 1.5 km long, at the neck of the peninsula making it a virtual ecological ‘island’. Fruit trees, notably cashew and marula, are grown in addition to large numbers of coconut palms. Some manioc and beans are cultivated in a limited area of the peninsula. A malaria control project was initiated in 2007. At the start of the project, all houses in the peninsula were mapped with handheld GPS units (Garmin e-Trex). Houses were numbered, photographed and their dimensions noted. Residents were informed of the purpose of the project and consented to participate.

Four hundred of the 467 houses enumerated in 2007 consisted of just a single room, 48 had two rooms and only 19 had more than two rooms. Most (87%) were built of either palm or reed and most had a gap between the roof and the walls, thus providing access for mosquitoes. Three-quarters of the houses had only one or two inhabitants. Half the households had a radio and 16% owned a mobile phone.

In a baseline malaria prevalence survey conducted in 2007, when no interventions were in use and there was no clinic on the peninsula, prevalence peaked at 35% in two to four-year olds, significantly lower than the 80% observed in the village of Furvela, across the bay. In addition to the 984 people enumerated in the initial census, the peninsula supported a population of circa 300 goats, 200 pigs and 26 dogs. Most people also kept chickens. Apart from a seasonal pond close to the middle of the peninsula and a permanent lake at its northern end, naturally occurring standing water is almost non-existent in Linga Linga. The water table is relatively high, however, and in the dry season, people dig shallow wells to obtain water for their crops.

### Entomology

**Figure 2:**
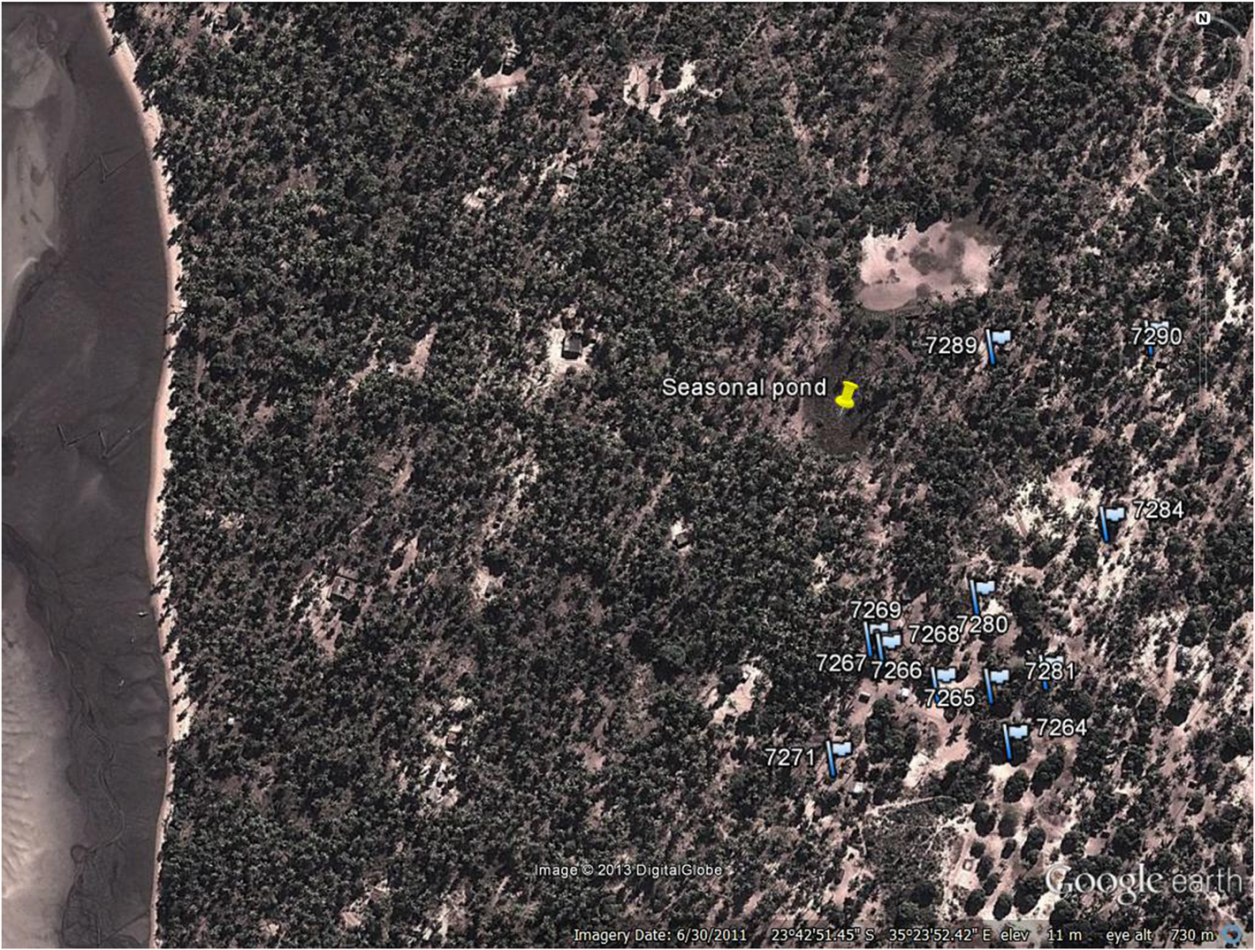
**Location of sampled houses in Linga Linga near the seasonal pond.** Numbers correspond to house identifiers listed in Table 2.

Two methods of collection for adult mosquitoes were used. Light-traps hung inside an occupied bedroom in which the occupant slept under a bed net were used for the collection of host-seeking females from 28 March 2009 (ISO week 13) to 24 May 2011 (ISO week 21). Exit collections, as described by Charlwood [21], were used to sample mosquitoes leaving houses at dusk from 25 May 2009 (ISO week 22) to 23 May 2011 (ISO week 21). Initial collections were undertaken in two areas of the peninsula and were subsequently concentrated in a group of houses close to the seasonal pond (Figure 2), a known breeding site. Thus, light-trap collections (from 16 April 2009 onwards) and exit collections (from 4 August 2009 onwards) were concentrated in an area expected to be one with a high density of vectors. Collected mosquitoes were categorized according to genus and as male or female. Females were further categorized according to abdominal status (unfed, part fed, semigravid or gravid). Anophelines were identified morphologically according to the keys of Gillies and De Meillon [19] and Gillies and Coetzee [22]. A subsample of the *An. funestus* collected during 2009 and stored over silica gel was tested for the presence of circumsporozoite protein in an enzyme linked immunosorbent assay (ELISA) using the protocols of Wirtz [23].

### Meteorology

For the entire study period, daily rainfall data from the town of Maxixe, approximately 15 km from Linga Linga, kindly provided by the Rio-Sul water management project, were used to compare mosquito numbers with environmental conditions. Also, for the year 2010, rainfall data were available from a digital weather station [24] which was established close to the southern tip of the peninsula with 360-degree exposure to wind.

### Statistical methods

Data was analysed in the statistical environment R [25]. Fisher’s exact test was used to calculate significance for rate ratios of abdominal states between seasons. The relationship between the mosquito density in houses and house characteristics (distance from pond and the number of inhabitants) was analysed using negative binomial regression with the number of mosquitoes collected as outcome variable, a logarithmic link function and the logarithmically transformed number of samples as offset [26].

### Ethical clearance

The project received ethical clearance from the National Bioethics Committee of Mozambique on 2August 2006 (reference 123/CNBS/06).

## Results

All mosquitoes classified as a member of the *An. funestus* group examined in detail had a single pale spot on the upper branch of the fifth vein and no pale spot at the tip of the sixth vein. In other words, they were morphologically all *An. funestus.* It appears reasonable to presume that it was the only member of the group being collected. Between 28 March 2009 and 24 May 2011, 3,684 *An. funestus* females and 91 males, 2,028 *Culex quinquefasciatus* females and 124 males, 67 *Mansonia* spp. and 106 unidentified culicines were collected during 961 light-trap nights and 790 female and 1,362 male *An. funestus* and 1,174 female and 1,026 male *Cx. quinquefasciatus*, 20 *Mansonia* spp. females and 22 unidentified culicines were caught during 391 exit collections undertaken in 59 houses, four of which were sampled more than 120 times. A comparison of mosquito densities in exit collections done during the period 25 May to 3 August 2009 showed that close to the tip of the peninsula, very few *An. funestus* were present as compared to in the middle of the peninsula (Table 1). The situation was inversed for *Culex* spp. A comparison of light-trap collections (over a much shorter period) was inconclusive. Subsequent analysis excludes data from houses near the tip of the peninsula.

**Table 1:** Comparison of collection results between the southern end and the middle of the peninsula

**Collection method**	**Exit (25 May to 3 August 2009)**		**Light traps (28 March to 15 April 2009)**	
**Zone**	**Tip**	**Middle**	**Tip**	**Middle**
n	56	47	12	5
*An. funestus* females	0.07	5.40	0.08	0.00
*An. funestus* males	0.02	6.57	0.00	0.00
*Culex* spp. females	5.09	1.87	18.17	29.60
*Culex* spp. males	1.04	3.43	0.42	0.00

Numbers indicate density per collection. The number of collections is indicated by n.

Figure 2 shows the location of the houses most commonly sampled relative to the seasonal pond and coast. Table 2 shows their collection results, including distance from the pond, and number of regular inhabitants. Table 3 shows results from negative binomial regression. In light-trap collections, *An. funestus* female density significantly decreased with distance from the pond, and increased with the number of inhabitants. The density of *An. funestus* males also decreased significantly with distance, but decreased with the number of inhabitants. *Culex* spp. density increased with inhabitants both in light-trap and exit collections, but was unrelated to distance from the pond.

**Table 2:** Collection results for the 12 most sampled houses

**House**	**Inhabitants**	**Distance from pond (m)**	**Light-trap collections**					**Exit collections**				
				***An. funestus***		***Culex *spp.**			***An. funestus***		***Culex *spp.**	
			**n**	**Females**	**Males**	**Females**	**Males**	**n**	**Females**	**Males**	**Females**	**Males**
7264	4	222	105	403	4	178	14	33	50	92	87	107
7280	1	134	97	201	36	124	8	25	48	42	55	63
7281	2	195	92	331	16	125	17	27	107	153	70	89
7267	4	135	82	654	1	241	15	47	125	164	143	163
7265	1	166	78	105	0	92	6	13	17	12	27	29
7289	3	96	73	97	1	130	6	14	14	64	36	62
7271	1	210	67	47	0	129	4	27	35	40	66	92
7266	2	183	63	214	6	44	1	12	58	123	44	40
7269	2	129	48	1215	4	92	14	24	120	337	55	38
7284	1	172	46	60	0	45	2	11	28	10	26	31
7268	1	139	26	48	4	34	6	14	54	208	20	31
7290	1	200	18	8	0	31	1	2	1	2	2	3

The number of collections is indicated by n.

**Table 3:** Regression coefficients of mosquito density depending on house characteristics

	**Light-trap collections**				**Exit collections**			
	***An. funestus***		***Culex *spp.**		***An. funestus***		***Culex *spp.**	
	**Females**	**Males**	**Females**	**Males**	**Females**	**Males**	**Females**	**Males**
Distance from pond (m)	−0.018**	−0.009*	−0.001	−0.005	−0.003	−0.011	0.000	0.001
Inhabitants	0.567**	−0.511***	0.157*	0.173	−0.008	0.069	0.090**	0.109

α: ***0.001, **0.01, *0.05.

**Figure 3:**
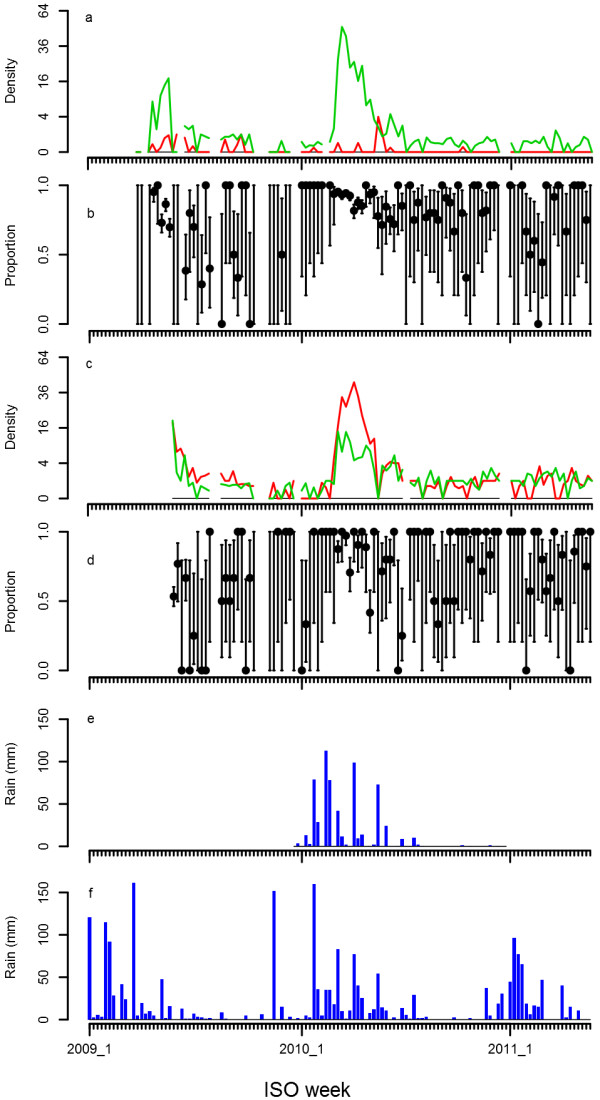
***Anopheles funestus *density, and proportion gravid and rainfall over the study period. a**) *An. funestus* density in light trap collections. The green line corresponds to females and the red line corresponds to males. **b**) Proportion of unfed *An. funestus* females in light trap collections. Black dots indicate the mean and bars indicate the 95% confidence interval. **c**) *An. funestus* density in exit collections. The green line corresponds to females and the red line corresponds to males. **d**) Proportion of unfed *An. funestus* females in exit collections. Black dots indicate the mean and bars indicate the 95% confidence interval. **e**) Weekly rainfall in mm measured in Linga Linga. **f**) Weekly rainfall in mm measured in Maxixe.

**Figure 4:**
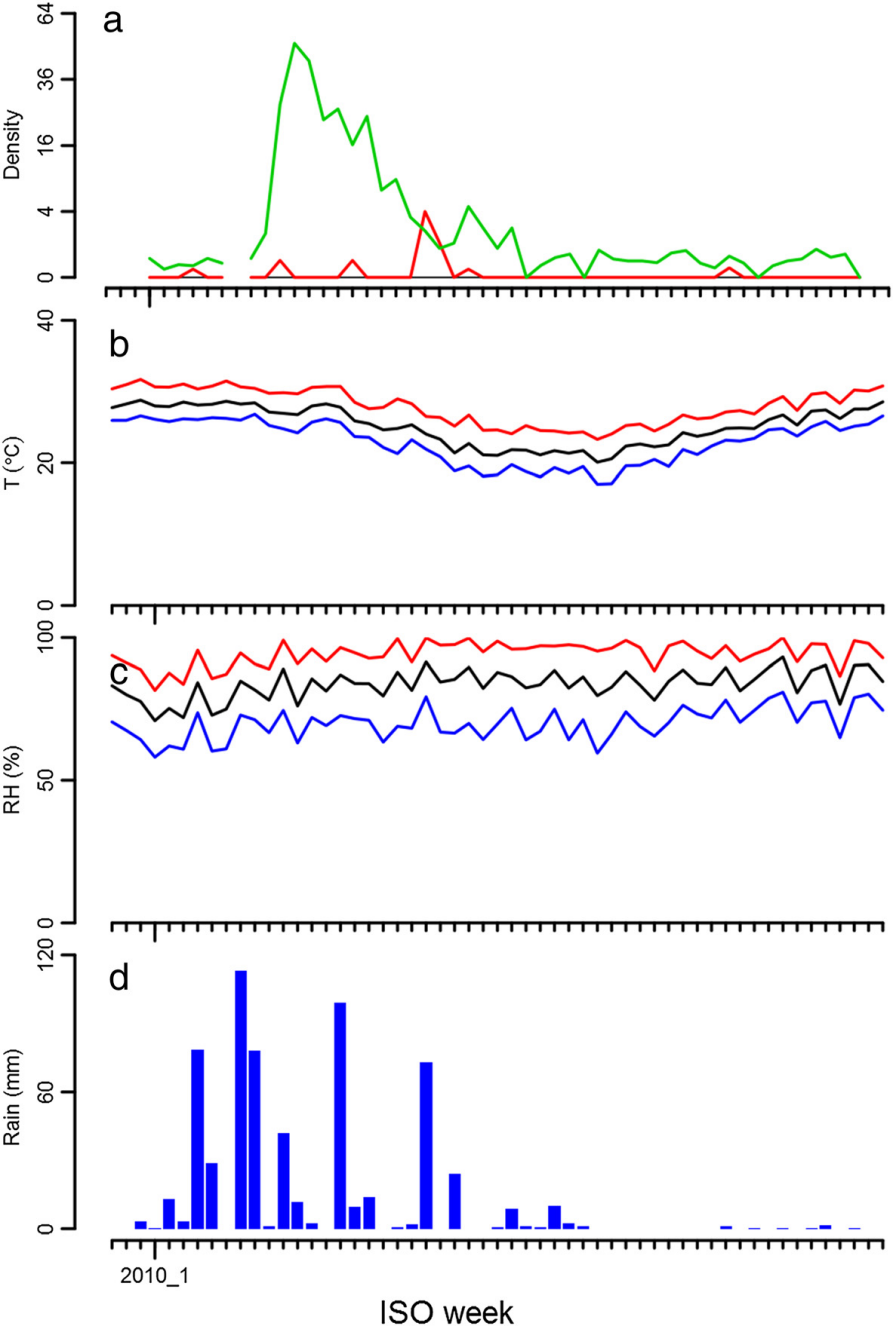
***Anopheles funestus *density in Linga Linga and weather by ISO week over 2010. a**) *An. funestus* density in light trap collections. The green line corresponds to females and the red line corresponds to males. **b**) Temperature (T) in degrees Celsius averaged by week. The red line corresponds to the daily maximum, the black to the daily mean, and the blue to the daily minimum. **c**) Per cent relative humidity (RH) averaged by week. The red line corresponds to the daily maximum, the black to the daily mean, and the blue to the daily minimum. **d**) Weekly rainfall in mm.

Figure 3 shows the weekly density of *An. funestus* collected using light traps (panel a), those exiting at dusk (panel c) on square root scales, and rainfall from Linga Linga (panel e) and Maxixe (panel f). In 2009 and 2010, a single annual peak in density coincident with the single annual peak in rainfall was observed. Curiously, a clear population peak was absent during the first 21 weeks of 2011. During the first half of 2010, rainfall measurements in Linga Linga showed a high correlation with those from Maxixe, but showed little rainfall towards the end of 2010 (see also Figure 4 for more detail), whereas rainfall was more substantial in Maxixe during that period. Light traps predominantly caught females, whereas many males were also caught while exiting houses at dusk. In between population peaks, *An. funestus* remained present at low densities. Figure 3 also shows weekly data on the unfed proportion of the female *An. funestus* collected in light-traps (panel b) and exiting houses at dusk (panel d). Despite the noisy nature of these data, the proportion of unfed females appears to increase with increasing density. Temperature and relative humidity stayed high throughout the year (Figure 4).

Table 4 shows the detailed information about the abdominal status of female *An. funestus* by season and by collection method. Peak season was defined as weekly density or three week moving average densities of over five females per sample, which corresponds to periods of ISO weeks 17 to 22 in 2009 and ISO weeks 9 to 18 in 2010. In light-trap collections, the proportion of gravid mosquitoes was significantly higher during low season than during the peak season (RR = 4.3, p<0.001). In contrast, in exit collections, the proportion of gravid mosquitoes was significantly lower during low season than during the peak season (RR = 0.64, p<0.01). Also, in light traps, the proportion of part-fed females was higher during the low season than during the peak season (RR = 4.5, p<0.001), whereas this was inversed for engorged females (RR = 0.46, p<0.05).

**Table 4:** **Comparison of the distribution of *Anopheles funestus *abdominal state between seasons depending on collection type**

**Type**	**Exit collection**						**Light-trap collection**					
**Season**	**Low**		**Peak**		**Season**	**Low**	**Peak**		**Season**		**Low**	**Peak**
	**%**	**n**	**%**	**n**	**RR**	**Sign**	**%**	**n**	**%**	**n**	**RR**	**Sign.**
Collections		85		11				86		16		
Unfed	75.5	219	71.6	355	1.06	NS	77.8	351	89.0	2876	0.87	***
Part fed	8.3	24	5.4	27	1.52	NS	2.7	12	0.6	19	4.53	***
Fed	2.1	6	1.8	9	1.14	NS	1.6	7	3.3	108	0.46	*
Semigravid	0.7	2	0.2	1	3.42	NS	3.1	14	3.6	117	0.86	NS
Gravid	13.4	39	21.0	104	0.64	**	14.9	67	3.5	112	4.29	***
Total	100.0	290	100.0	496			100.0	451	100.0	3232		
												
Part fed + fed	10.3	30.0	7.3	36.0	1.43	NS	4.2	19.0	3.9	127.0	1.07	NS
Semi-gravid + gravid	14.1	41.0	21.2	105.0	0.67	*	18.0	81.0	7.1	229.0	2.53	***

n: count; RR: rate ratio; Sign.: significance in Fisher’s exact test; α: ***0.001, **0.01, *0.05, NS 1.

Overall, female *An. funestus* were significantly more often part-fed (RR = 2.1, p<0.001) and semigravid (RR = 2.2, p<0.001) in exit collections than in light-trap collections (Table 5).

**Table 5:** **Comparison of the distribution of *Anopheles funestus *abdominal state between collection types depending on season**

**Season**	**Low**						**Peak**						**All**	
**Type**	**Exit collection**		**Light-trap collection**				**Type**		**Exit collection**		**Light-trap collection**			**Type**
	**%**	**n**	**%**	**n**	**RR**	**Sign.**	**%**	**n**	**%**	**n**	**RR**	**Sign.**	**RR**	**Sign.**
Collections		85		86				11		16				
Unfed	75.5	219	77.8	351	0.97	NS	71.6	355	89.0	2876	0.80	***	0.83	***
Part fed	8.3	24	2.7	12	3.11	***	5.4	27	0.6	19	9.26	***	7.71	***
Fed	2.1	6	1.6	7	1.33	NS	1.8	9	3.3	108	0.54	NS	0.61	NS
Semigravid	0.7	2	3.1	14	0.22	*	0.2	1	3.6	117	0.06	***	0.11	***
Gravid	13.4	39	14.9	67	0.91	NS	21.0	104	3.5	112	6.05	***	3.74	***
Total	100.0	290	100.0	451			100.0	496	100.0	3232				
Part fed + fed	10.3	30.0	4.2	19.0	2.46	***	7.3	36.0	3.9	127.0	1.85	**	2.12	***
Semi-gravid + gravid	14.1	41.0	18.0	81.0	0.79	NS	21.2	105.0	7.1	229.0	2.99	***	2.21	***

n: count; RR: rate ratio; Sign.: significance in Fisher’s exact test; α: ***0.001, **0.01, *0.05, NS 1.

Thirteen out of 289 (4.5%) *An. funestus* tested positive in the sporozoite ELISA, five being high density infections and eight being low density ones. The proportion of sporozoite-positive females was higher during the low season (6.25%, six out of 96) than during the peaks (3.63%, seven out of 193), but this difference was not significant (Fisher’s exact test, α=0.05).

## Discussion

Although the *An. funestus* were not identified to species by polymerase chain reaction, given that all specimens examined in detail morphologically keyed out to this member of the group, its endophilic behaviour and the sporozoite rate observed, it seems reasonable to assume that most, if not all, mosquitoes identified in Linga Linga were *An. funestus*. Two population density peaks were observed over a 104-week period of collection, both occurring within the first 21 weeks of the year, during and following the rainy season. With relative humidity and temperature being favourable throughout the year, rather than being driven by temperature (as occurs in a number of other *An. funestus* populations [19]) rainfall apparently determines the population dynamics on Linga Linga. However, during the first 21 weeks of a third year, a clear population density peak was absent. Despite some apparent association of adult density in houses with rainfall and with proximity to a seasonal pond (in which anopheline larvae were found), breeding in other places (such as the shallow wells dug during the dry season) cannot be excluded. Such breeding sites could be responsible for the low but continued presence of *An. funestus* during the dry season. The fact that in Linga Linga, males continued to be present in exit collections during the dry season could also indicate continued breeding, but with some sources of nectar available, it is also possible that the males will survive as long as females. By contrast, in the Sahel, male mosquitoes disappear during the dry season, since none of the plants that might be a source of sugar blossom during that period [7]. Mosquito populations were not investigated close to the permanent lake at the northern end of the peninsula and it is possible that some immigration from this site occurred in the study area, migration of older mosquitoes from Furvela, 8 km across the bay, was also possible though unlikely due to the wide natural barrier of salt water. Otherwise, the village of Furvela is bordered on two sides by the floodplains of the Furvela and Ngombe rivers, both of which are perennial. Breeding continues throughout the year in Furvela and is not associated with rainfall.

The higher proportions of gravid and partially fed females in light traps during the low season compared to during the peak season, coupled with lower proportions of unfed females, indicate that during the low season, proportionally more gravid and partially fed females may be searching for a blood meal. This cannot easily be explained by the possibility of there simply being more gravid (and less virgin) females around during the low season, because in exit collections at dusk, the proportion of gravid females was lower during the low season than during the peak season. In the absence of interventions, such as bed nets, when most host-searching females manage to feed, exit collections at dusk were typically dominated by gravid mosquitoes in search of an oviposition site, and virgin mosquitoes in search of a mate (19). In Linga Linga, where bed nets were used, as in the sentinel houses, the proportion of host-searching females that failed to find a host the previous night may be somewhat larger (20). Nevertheless, even if there were proportionally more gravid mosquitoes during the low season (as indicated by the results from the light-trap collections), then these endophilic mosquitoes might not be exiting from houses to oviposit (due to a lack of breeding sites) but instead linger longer around or in houses, and presumably, when hungry, will still feed on people, hence the relatively low proportion of gravid females in exit collections.

## Conclusions

During the rainy season, the *An. funestus* population in Linga Linga is a typically gonotrophically concordant one (with high densities of unfed females in light-trap collections and gravid ones in exit collections). However, the analysis suggests gonotrophic discordance as a possible explanation for the observed abdominal status distributions in collections during the dry season. Unfortunately, no dissections were done to establish mating status, and ovary status, which could support such an explanation. The observed sporozoite rate of 4.5%, in the presence of a relatively low infection prevalence in humans who predominately use bed nets, also implies a high mosquito life expectancy. Despite the low numbers of mosquitoes collected, positive cases of malaria (determined by microscopy and/or rapid test) occurred throughout the year [Charlwood, unpublished observations]. Therefore, for periods when breeding sites are scarce, it is suggested that such malaria transmission in Linga Linga may be largely sustained by a small number of long-lived, gonotrophically discordant, females.

## Competing interests

The authors declare that they have no competing interests.

## Authors’ contributions

JDC conceived of the study, and wrote the manuscript; NC helped with logistics and the study design; EVET helped with mosquito collection and performed the ELISA; OJTB performed statistical analysis, and wrote the manuscript. All authors read and approved the final manuscript.
